# Effects of private health insurance on medical expenditure and health service utilization in South Korea: a quantile regression analysis

**DOI:** 10.1186/s12913-023-10251-x

**Published:** 2023-11-07

**Authors:** Kristine Namhee Kwon, Wankyo Chung

**Affiliations:** 1 Department of Health Policy and Management, Milken Institute School of Public Health, George Washington University, Washington, DC USA; 2 Department of Public Health Sciences, Graduate School of Public Health, Seoul National University, Seoul, South Korea

**Keywords:** Private health insurance, Medical expenditure, Moral hazard, Quantile regression, Quantile count regression, Propensity score matching

## Abstract

**Background:**

Despite universal health insurance, South Korea has seen a sharp increase in the number of people enrolled in supplemental private health insurance (PHI) during the last decade. This study examined how private health insurance enrollment affects medical expenditure and health service utilization.

**Methods:**

Unbalanced panel data for adults aged 19 and older were constructed using the 2016–2018 Korea Health Panel Survey. Quantile regression for medical cost, and quantile count regression for health service utilization were utilized using propensity score-matched data. We included 17 variables representing demographic, socioeconomic, and health information, as well as medical costs and use of outpatient and inpatient care.

**Results:**

We discovered that PHI enrollees’ socioeconomic and health status is more likely to be better than PHI non-enrollees’. Results showed that private health insurance had a greater effect on the lower quantiles of the conditional distribution of outpatient costs (coefficient 0.149 at the 10th quantile and 0.121 at the 25th quantile) and higher quantiles of inpaitent care utilization (coefficient 0.321 at the 90th quantile for days of hospitalization and 0.076 at the 90th quantile for number of inpatient visits).

**Conclusions:**

PHI enrollment is positively correlated with outpatient costs and inpatient care utilization. Government policies should consider these heterogeneous distributional effects of private health insurance.

**Supplementary Information:**

The online version contains supplementary material available at 10.1186/s12913-023-10251-x.

## Introduction

The South Korean government successfully implemented universal public health insurance in 1989, only 12 years after the commencement of national health insurance in 1977, substantially improving access to healthcare. Nevertheless, despite the government’s ongoing efforts over the last 20 years, national health insurance coverage has increased marginally from 62.5% in 2012 to 65.3% in 2020 [see Additional file 1: Appendix 1]. This coverage rate is remarkably low compared to the OECD average, with 73.8% of total health spending covered by government or compulsory insurance [1]. Although over 97% of South Koreans are covered by national health insurance, they enroll in private health insurance (PHI) as a supplementary means to cover medical expenses not covered by national health insurance. Increasing enrollment rates resulted from rapid aging, the prevalence of chronic diseases, income growth, and the advancement of modern health technology [2, 3]. PHI enrollment rate per household rose rapidly from 69.8% in 2012 to 78.6% in 2020 [see Additional file 1: Appendix 1]. Simultaneously, there has been a growing concern that PHI enrollees will use health services excessively, which would severely impact the nation’s health financing.

There has been an interaction between PHI and public health insurance [4]. The PHI plays a positive supplemental role in supporting medical services not covered by public insurance. It ensures that patients have a wider range of options, disperses the risk among enrollees, and introduces more resources and funding into the healthcare system [5]. Conversely, there exist concerns about the potential excessive utilization of health services caused by PHI [6]. Therefore, it is crucial to amass empirical evidence regarding the effects of PHI to potentially mitigate the excessive utilization of health services [5, 7].

The conditional mean of outcome variables was used in the majority of the earlier studies. However, medical expenditure and health service utilization have a positive skewness with a long tail to the right and typically have many zero values [8]. As a result, it is critical to consider these data characteristics and the heterogeneous effect of PHI on the conditional distribution of outcome variables. This study utilizes quantile regression to examine the effects of PHI enrollment on the conditional distributions of medical expenditure and health service utilization.

### Moral hazard and adverse selection

Moral hazard and adverse selection are frequently employed to explain how enrollment in PHI has raised medical expenditure and health service utilization. According to Pauly (1968), moral hazard refers to a situation in which a person uses more health services because their marginal health costs are reduced after purchasing PHI [9].

Empirical analysis on whether PHI engenders moral hazard has been steadily accumulating in Korea. Previous studies presented different results depending on the data source, study period, target population, and PHI characteristics. Overall, PHI enrollment significantly increased outpatient costs [2, 10–12] and services [2, 7, 10, 13–16]. Different results were presented by PHI type that indemnity insurance increased the days of hospitalization and the number of inpatient visits, while fixed benefit insurance increased only binary indicator for the use of inpatient service [7, 10]. When classified by gender, indemnity insurance did not affect males’ cost per hospitalization but increased females’ [17]. When classified by initial enrollment of PHI, it increased not only outpatient visits and medical check-up visits but also significantly increased the days of hospitalization [15]. Earlier studies showed the existence of moral hazard [2, 10, 12–14], which was more evident in those who enrolled in more than two PHIs [13] and those with higher incomes [14].

Adverse selection refers to a phenomenon in which those who are more likely to incur costs, such as older adults or people with diseases, are more likely to self-select into purchasing insurance. In contrast, favorable selection (also known as advantageous or propitious selection) refers to a phenomenon in which individuals who make more preventative efforts to maintain good health will self-select into purchasing insurance [18].

Empirical research findings in Korea showed a mix of adverse and favorable selection. It was reported that socially vulnerable groups, including low-income families, older adults, and the disabled had a low likelihood of enrolling in PHI [3] and older adults as well as those with chronic diseases or disabilities were less likely to be enrolled in indemnity insurance [19]. Furthermore, the enrollment rate of older adults into PHI was higher among those with fewer chronic diseases and poor self-rated health [20].

Individual and insurer selection can influence the decision to purchase PHI, causing potential sources of endogeneity. Previous literature has attempted to address this issue using instrument variables [10, 21, 22], panel data analysis [15–17], and propensity score matching [2, 11, 23, 24]. An additional file summarizes this in more detail [see Additional file 1: Appendix 2]. We utilized propensity score matching.

## Methods

### Data and sample

We used the Korea Health Panel Survey (KHPS) data from 2016 to 2018, provided by Korea Institute for Health and Social Affairs (KIHSA) and National Health Insurance Service (NHIS). The data provides detailed information on health service utilization, socioeconomic characteristics, health status, and health behavior (https://www.khp.re.kr:444/eng/main.do).

The study population consists of adults aged 19 years or older, excluding younger populations whose parents will decide their enrollment in PHI. We examined a total of 38,074 person-years: 12,701 in 2016, 12,692 in 2017, and 12,681 in 2018.

### Variables

The main independent variable is PHI enrollment, and the dependent variables are medical expenditure and health service utilization. Three medical expenditure variables are outpatient, inpatient, and total expenditures. Total expenditure is the sum of outpatient and inpatient expenditures. Three health service utilization variables are the number of outpatient visits, inpatient visits, and days of hospitalization. An additional file shows a detailed description of the variables [see Additional file 1: Appendix 3]. Other control variables were selected based on Andersen’s behavioral model [25]. Predisposing factors included gender, age, educational level, and marital status. Residence, type of health insurance enrollment, economic activity status, and annual income were used as enabling factors. Finally, for illness-level factors, self-rated health, the number of diagnosed chronic diseases, and Charlson’s comorbidity index (CCI) were used. The CCI calculation is based on previous literature [26, 27]. An additional file shows more detail on the CCI calculation using the KHPS [see Additional file 1: Appendix 4].

### Propensity score matching

The unobserved heterogeneity between PHI enrollees and non-enrollees may lead to selection bias [14]. A control group (PHI non-enrollees) with similar attributes to the treated group (PHI enrollees) was chosen to moderate this bias. Observable factors such as age, income level, chronic disease, and unobservable factors such as perception of health risks and preference for health service use may have multi-dimensional effects on the decision to purchase PHI. This study uses probit to estimate the propensity score and likelihood of enrolling in PHI. The estimation model is as follows.

$$\text{Pr}\left({Y}_{i}=PHI\right)={\beta }_{0}+{\beta }_{1}{Z}_{i}+{\beta }_{2}{H}_{i}+{\varphi }_{i}$$

${Z}_{i}$ refers to socioeconomic characteristics such as gender, age, education level, marital status, residential area, type of insurance, economic activity, and yearly income quantile. ${H}_{i}$ denotes health status such as self-rated health, number of diagnosed chronic diseases, and Charlson’s comorbidity index. Nearest-neighbor matching was used without replacement.

### Quantile regression

Medical expenditure variables such as outpatient, inpatient, and total costs were analyzed using quantile regression. The quantile regression analysis can be expressed as follows [28].

$$\text{Q}\left({\beta }_{q}\right)=\sum _{i:{y}_{i}\ge {x{\prime }}_{i}\beta }^{N}q|{y}_{i}-{x{\prime }}_{i}{\beta }_{q}|+\sum _{i:{y}_{i}<{x{\prime }}_{i}\beta }^{N}(1-q)|{y}_{i}-{x{\prime }}_{i}{\beta }_{q}|$$

Quantile regression estimates the coefficient ${\beta }_{q}$ that minimizes the sum of the absolute value of the negative residual weighted by $\left(1-\text{q}\right)$ and the positive residual weighted by $\left(\text{q}\right)$, through linear programming [28], where $\text{q}$ denotes quantiles between 0 and 1.

While many studies used conditional mean of the outcome variable, quantile regression provides a more complete view of the relationship between the dependent and independent variables along the conditional distribution of the dependent variable [28, 29]. When dependent variables such as medical cost and health service utilization do not follow a normal distribution, or when outliers are present, quantile regression is preferable as an alternative method. Furthermore, a non-parametric approach is also possible since the distributional assumption for the regression residuals has not been made. Because of these methodological benefits, quantile regression has been actively used [30–32] and quantile regression utilizing instrumental variables [33], panel data [34], or additive data [35–39] have also been actively used.

### Quantile count regression

Existing methodologies dealing with count data have limitations in assuming the distribution of the dependent variable and presenting results only about the conditional mean. Quantile count regression, on the other hand, makes no assumptions about the dependent variable’s distribution and has the advantage of examining conditional quantiles [37, 40, 41]. For the conditional quantile to be estimated, the dependent variable must be continuous for the optimization problem to be solved. Machado and Silva (2005) developed quantile regression for count data by introducing a jittering procedure for discrete dependent variables such as additive data [36–41].

We used quantile count regression to examine health service utilization: number of outpatient visits, inpatient visits, and days of hospitalization. Due to overdispersion, a negative binomial regression model was chosen over the Poisson model. For example, we had zero outpatient and inpatient visits/hospitalization days for 18.5% and 87.3% of our samples, respectively. Thus, negative binomial regression was used for the former, and zero-inflated negative binomial regression for the latter. We provided both marginal effects as well as coefficient estimates.

## Results

### Descriptive statistics

Table 1 summarizes the characteristics of the study population throughout the three years. The PHI enrollment rate increased gradually from 73.0% to 2016 to 74.4% in 2017 to 75.6% in 2018 for Korean individuals aged 19 or older. The PHI enrollment rate for the whole population was 74.3%. The sample was comprised of female (54.9%), college or higher education (37.9%), married (68.8%), enrolled in NHI (96.7%), and economically active (61.1%). The average age was 54 years and the average income was $12,797. As for health-related variables, 15.8% reported poor self-rated health, an average of 2.1 diagnosed chronic conditions, and a 0.350 CCI score.

**Table 1:** Characteristics of the study population

Variables	2016 (N = 12,701)		2017 (N = 12,692)		2018 (N = 12,681)		Whole sample (N = 38,074)	
	**Mean**	**S.D.** ^c^	**Mean**	**S.D.** ^c^	**Mean**	**S.D.** ^c^	**Mean**	**S.D.** ^c^
*Independent variable*								
PHI enrollment	0.730	0.444	0.744	0.436	0.756	0.429	0.743	0.437
*Outcome variables*								
Outpatient exp. ($)^a^	545.40	1176.017	575.95	1187.105	621.90	1230.292	581.07	1198.400
Inpatient exp. ($)^a^	466.97	2501.890	453.79	2156.125	519.61	2597.902	480.11	2426.138
Total exp. ($)^a^	1012.37	3028.710	1029.74	2723.133	1141.51	3096.813	1061.18	2954.477
No. of outpatient visits	19.1	27.158	19.4	26.725	20.2	27.046	19.6	26.980
No. of inpatient visits	0.5	4.849	0.5	6.454	0.6	5.206	0.5	5.546
Days of hospitalization	1.8	9.885	1.8	9.855	1.9	9.581	1.8	9.775
*Control variables*								
Female	0.550	0.498	0.550	0.498	0.548	0.498	0.549	0.498
Age	53.711	17.084	54.027	17.312	54.341	17.614	54.026	17.339
Educational level								
Elementary or less	0.209	0.406	0.204	0.403	0.200	0.400	0.204	0.403
Junior high	0.112	0.315	0.110	0.313	0.106	0.308	0.110	0.312
Senior high	0.310	0.463	0.307	0.461	0.305	0.461	0.308	0.461
Above college	0.369	0.482	0.379	0.485	0.389	0.487	0.379	0.485
Married	0.699	0.459	0.688	0.463	0.678	0.467	0.688	0.463
Reside in Capital^b^	0.373	0.484	0.374	0.484	0.378	0.485	0.375	0.484
NHI enrollment	0.965	0.183	0.967	0.178	0.968	0.175	0.967	0.179
Economically active	0.604	0.489	0.611	0.488	0.619	0.486	0.611	0.487
Yearly income ($)	12130.47	10532.93	12771.64	8843.49	13490.02	8890.36	12797.02	9471.42
Poor self-rated health	0.157	0.364	0.158	0.365	0.160	0.366	0.158	0.365
No. of chronic diseases	2.053	2.343	2.124	2.388	2.218	2.465	2.132	2.400
CCI scores	0.347	0.762	0.333	0.726	0.369	0.778	0.350	0.756

Note: ^a^1USD ≈ 1,000KRW; ^b^Capital cities refers to Seoul, Incheon, and Gyeonggi-do; ^c^S.D. (Standard deviation); exp. (expenditure); No. (Number); NHI. (National Health Insurance)

**Fig. 1 Distributions of outcome variables without zero values:**
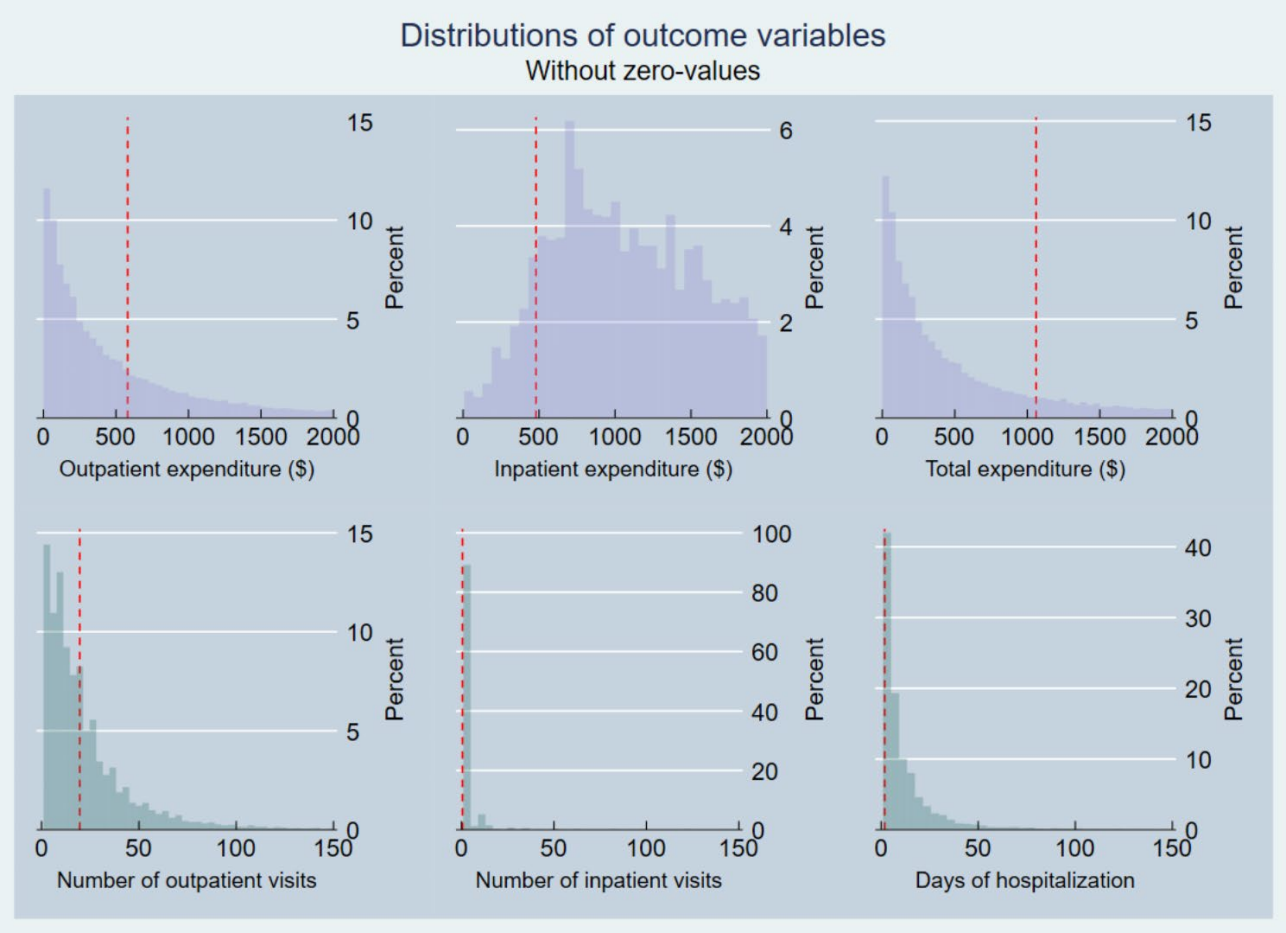
Figure 1 displays distributions of outcome variables without zero values. As shown, medical expenditure and health service utilization have significant point mass at zero and skewness with long right tails. These necessitate a study on conditional quantiles of outcome variables. Apart from inpatient expenditure, they are densely populated at the lower quantiles. Note: Top row displays expenditures less than $2,000 conditional on positive values. The red dashed line represents the mean of each variable. 18.5% of outpatient expenditure equals to zero-values, 87.3% of inpatient expenditure equals to zero-values, and 18.2% of total expenditure equals to zero-values. Similarly, utilization of fewer than 100 counts conditional on positive values is displayed on the bottom row. 18.5% of outpatient visits equal to zero-values, 87.3% of inpatient visits and inpatient days equal to zero-values.

### Matching result

**Fig. 2:**
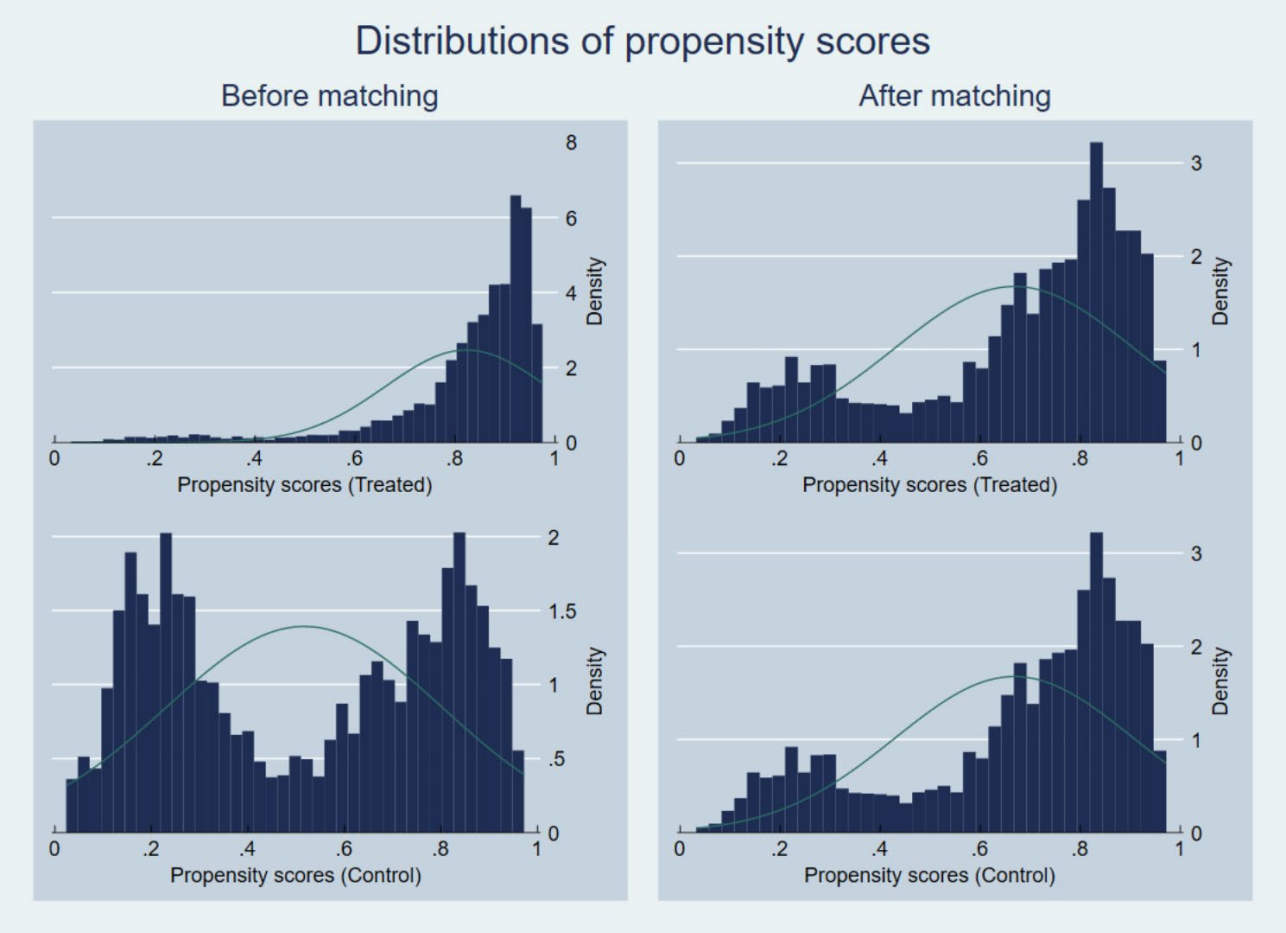
Distributions of propensity scores before and after matching

When matching is used for endogenous PHI enrollment, the quality of matching can be evaluated by balancing test and common support conditions [42]. Table 2 shows the Chi-square test after matching and the results revealed no significant difference between treated and control groups for any covariates used. PHI enrollees are more likely to be well-educated, married, economically active, have national health insurance, and have higher incomes. As for health, PHI enrollees are more likely to have better self-rated health, lower CCI scores, and fewer diagnosed chronic diseases. Moreover, propensity score distributions were confirmed to be different before and after matching, as shown in Fig. 2.

**Table 2:** Characteristics of the pooled population before and after matching

Variables	Before matching (n = 38,074)				*p* ^d^	After matching (n = 10,654)				*p* ^d^
	**PHI** ^a^ **(n = 28,303)**		**Non-PHI** ^b^ **(n = 9771)**			**PHI** ^a^ **(n = 5327)**		**Non-PHI** ^b^ **(n = 5327)**		
**Gender**					0.159					0.846
Female	15,605	(55.1)	5307	(54.3)		2852	(53.5)	2862	(53.7)	
Male	12,698	(44.9)	4464	(45.7)		2475	(46.5)	2465	(46.3)	
**Age group**					p < 0.001					0.909
20–29	3302	(11.7)	640	(6.6)		568	(10.7)	576	(10.8)	
30–44	6788	(24.0)	1000	(10.2)		846	(15.9)	847	(15.9)	
45–59	9909	(35.0)	1170	(12.0)		858	(16.1)	821	(15.4)	
60–74	7009	(24.8)	2773	(28.4)		2080	(39.1)	2102	(39.5)	
75 +	1295	(4.6)	4188	(42.9)		975	(18.3)	981	(18.4)	
**Education**					p < 0.001					
Elementary	3544	(12.5)	4227	(43.3)		1679	(31.5)	1669	(31.3)	
Junior high	2922	(10.3)	1249	(12.8)		654	(12.3)	654	(12.3)	
Senior high	9482	(33.5)	2228	(22.8)		1360	(25.5)	1373	(25.8)	
Above college	12,355	(43.7)	2067	(21.2)		1634	(30.7)	1631	(30.6)	
**Marital status**					p < 0.001					0.920
Married	20,681	(73.1)	5531	(56.6)		3348	(62.9)	3343	(62.8)	
Single	7622	(26.9)	4240	(43.4)		1979	(37.2)	1984	(37.2)	
**Residence**					p < 0.001					0.788
Capital	11,126	(39.3)	3148	(32.2)		1738	(32.6)	1751	(32.9)	
Other areas	17,177	(60.7)	6623	(67.8)		3589	(67.4)	3576	(67.1)	
**Health insurance**					p < 0.001					0.538
NHI^c^	27,899	(98.6)	8915	(91.2)		5187	(97.4)	5197	(97.6)	
Medical Aid	404	(1.4)	856	(8.8)		140	(2.6)	130	(2.4)	
**Economically active**					p < 0.001					0.876
Yes	19,303	(68.2)	3996	(40.6)		2897	(54.4)	2889	(54.2)	
No	9000	(31.8)	5805	(59.4)		2430	(45.6)	2438	(45.8)	
**Yearly income**					p < 0.001					0.939
1Q (Low)	4744	(16.8)	4783	(49.0)		1874	(35.2)	1686	(35.1)	
2Q	7147	(25.3)	2363	(24.2)		1520	(28.5)	1498	(28.1)	
3Q	8030	(28.4)	1489	(15.2)		1043	(19.6)	1064	(20.0)	
4Q (High)	8382	(29.6)	1136	(11.6)		890	(16.7)	897	(16.8)	
**Self-rated Health**					p < 0.001					0.269
Bad	3186	(11.3)	2843	(29.1)		992	(18.6)	948	(17.8)	
Good	25,117	(88.7)	6928	(70.9)		4335	(81.4)	4379	(82.2)	
**Chronic diseases** ^**e**^					p < 0.001					0.954
0	11,044	(39.0)	1900	(19.5)		1635	(30.7)	1628	(30.6)	
1	5781	(20.4)	1182	(12.1)		646	(12.1)	656	(12.3)	
2 +	11,478	(40.6)	6689	(68.5)		3046	(57.2)	3043	(57.1)	
**CCI** ^**e**^					p < 0.001					0.938
0	23,247	(82.1)	5993	(61.3)		3808	(71.5)	3802	(71.4)	
1	3442	(12.2)	2283	(23.4)		979	(18.4)	974	(18.3)	
2 +	1614	(5.7)	1495	(15.3)		540	(10.1)	551	(10.3)	

Note: ^a^PHI (Private health insurance enrollees); ^b^Non-PHI (Private health insurance non-enrollees); ^c^NHI (National Health Insurance); ^d^Number in parentheses refers to the percentage (%), and p-value for ${\chi }^{2}$ is shown; ^e^The treated (PHI enrollees) had an average of 1.7 chronic diseases and a CCI of 0.3, while the control had 3.3 and 0.6, respectively

### PHI effects on medical expenditure

Table 3 shows descriptive statistics for medical expenditure, some with zero values and others without, to show their differences. PHI non-enrollees spend more on all types of medical expenditures, whether or not they include zero values. When zero-values are left out, average inpatient expenditure increases by 8.7 times for PHI enrollees and 6.1 times for others.

**Table 3:** Summary statistics of medical expenditure by PHI enrollment

Unmatched data	PHI^a^ (N = 28,303)				Non-PHI^b^ (N = 9771)			
	**Mean**	**S.D.** ^c^	**Min**	**Max**	**Mean**	**S.D.** ^c^	**Min**	**Max**
**0 included**								
Outpatient ($)	533.0	1148.881	0	35246.5	720.3	1321.713	0	23219.8
Inpatient ($)	393.9	2149.120	0	94645.0	730.0	3078.032	0	116260.5
Total ($)	926.8	2702.783	0	104238.3	1450.3	3556.809	0	119236.6
**0 not included**								
Outpatient ($)	660.7	1245.728	0.8	35246.5	857.9	1400.922	0.8	23219.8
Inpatient ($)	3452.2	5471.2	7.7	94645.0	4438.5	6416.1	92.6	116260.5
Total ($)	1145.7	2963.0	0.8	104238.3	1721.7	3814.6	0.8	119236.6

Note: ^a^PHI (Private health insurance enrollees); ^b^Non-PHI (Private health insurance non-enrollees); ^c^S.D. (Standard deviation)

Table 4 shows the effects of PHI enrollment on medical expenditure using matched data. As shown in Table 4, quantile regression provides more generic information than OLS regression. The OLS regression coefficient (0.082) is located between the 25th (0.121) and 50th (0.061) quantiles of the conditional distribution for outpatient expenditure. PHI enrollment showed statistically significant effects in the 10th and 25th quantiles of the distribution. The effect size decreases as the quantiles move up the distribution and become insignificant.

However, PHI enrollment showed no significant effect on inpatient expenditure in both OLS and quantile regressions. Even the direction of effect changes across the distribution, which can be better explained when separate regressions were used for male and female subsamples [see Additional file 1: Appendix 6]. While PHI enrollment increases female inpatient expenditure consistently, it decreases male inpatient expenditure, though not significantly. Thus, PHI enrollment may offset inpatient expenditure between males and females. Overall, PHI enrollment significantly increased total medical expenditure in all quantiles except the 90th quantile. The effect size declines across the 10th to 90th quantiles of conditional expenditure.

**Table 4:** Effects of PHI enrollment on the distribution of medical expenditure

Matched data	OLS regression	Quantile regression						
		0.10	0.25	0.50	0.60	0.70	0.80	0.90
**Outpatient**	0.082^**^	0.149^**^	0.121^***^	0.061	0.043	0.022	0.028	0.038
(N = 8616)	(0.026)	(0.047)	(0.036)	(0.031)	(0.030)	(0.032)	(0.029)	(0.037)
**Inpatient**	-0.044	-0.018	0.021	-0.017	-0.016	-0.056	0.016	0.040
(N = 1470)	(0.056)	(0.051)	(0.072)	(0.065)	(0.079)	(0.074)	(0.058)	(0.084)
**Total**	0.111^***^	0.192^***^	0.159^***^	0.101^**^	0.085^*^	0.089^*^	0.103^*^	0.082
(N = 8634)	(0.031)	(0.041)	(0.038)	(0.037)	(0.038)	(0.038)	(0.045)	(0.051)

Note: Numbers in parentheses represent the robust standard errors. Other covariates were not displayed on the table. ^*^p < 0.05, ^**^p < 0.01, ^***^p < 0.001

Other significant control variables for the outpatient cost were age, the number of chronic diseases, being married, and poor self-reported health. The number of chronic diseases and age showed greater impact at the lower quantiles of outpatient cost, while being married and poor self-rated health did at the 50th percentile. Meanwhile, both poor self-rated health and CCI significantly influenced the higher quantiles of inpatient costs [see Additional file 1: Appendix 5].

### PHI effects on health service utilization

Table 5 presents descriptive statistics for health service utilization, some with zero values and others without, to show their differences. PHI non-enrollees use more on all types of medical services, whether or not they include zero values. When zero-values are left out, average inpatient days increase by 8.8 times for PHI enrollees and 6.1 times for others.

**Table 5:** Summary statistics of health service utilization by PHI enrollment

Unmatched data	PHI^a^ (N = 28,303)				Non-PHI^b^ (N = 9771)			
	**Mean**	**S.D.** ^c^	**Min**	**Max**	**Mean**	**S.D.** ^c^	**Min**	**Max**
**0 included**								
Outpatient visit	16.65	23.093	0	361	28.03	34.577	0	348
Inpatient visit	0.45	5.565	0	361	0.74	5.483	0	256
Inpatient days	1.44	8.410	0	304	2.92	12.876	0	299
**0 not included**								
Outpatient visit	20.65	24.056	0	361	33.39	35.287	1	348
Inpatient visit	3.91	16.062	1	361	4.50	12.885	1	256
Inpatient days	12.62	21.888	1	304	17.77	27.239	1	299

Note: ^a^PHI (Private health insurance enrollees); ^b^Non-PHI (Private health insurance non-enrollees); ^c^S.D. (Standard deviation)

Table 6 shows the effects of PHI enrollment on health service utilization using quantile count regression. Because quantile count regression employs a jittering procedure to smooth the outcome variables, marginal effects and elasticities are used to explain the results [41]. Table 6 presents marginal effects at the mean.

The marginal effects show a positive and statistically significant effect of PHI enrollment on outpatient visits. The effect size increases from the 10th to 50th quantiles but declines thereafter. As for inpatient services, the positive effect of PHI enrollment increases to 90th quantiles. Interestingly, it shows a greater effect on inpatient days than on the number of inpatient visits. When separate regressions were used for male and female subsamples, PHI enrollment increased male inpatient visits and days of hospitalization, though not statistically significant [see Additional file 1: Appendix 6]. 

**Table 6:** Marginal effects of PHI enrollment on the distribution of health service utilization

Matched data	NB /ZINB^a^	Quantile count regression						
		0.10	0.25	0.50	0.60	0.70	0.80	0.90
**Outpatient visits**	2.283^***^	0.470^***^	0.873^***^	1.825^***^	1.648^***^	1.280^**^	1.478^*^	0.407
(N = 10,654)	(0.581)	(0.076)	(0.118)	(0.245)	(0.339)	(0.465)	(0.648)	(0.995)
**Inpatient visits**	0.109	0.004^*^	0.006^***^	0.013^***^	0.017^***^	0.020^***^	0.028^**^	0.076^**^
(N = 10,654)	(1.136)	(0.002)	(0.002)	(0.004)	(0.005)	(0.006)	(0.009)	(0.029)
**Inpatient days**	0.045	0.004^*^	0.006^***^	0.013^***^	0.017^***^	0.024^**^	0.050^*^	0.321^**^
(N = 10,654)	(0.140)	(0.002)	(0.002)	(0.004)	(0.005)	(0.008)	(0.019)	(0.119)

Note: ^a^Negative binomial regression (NB) is used for outpatient visits, and Zero-inflated negative binomial regression (ZINB) is used for inpatient visits and inpatient days. Numbers in parentheses represent the robust standard errors. Other covariates were not displayed on the table. ^*^p < 0.05, ^**^p < 0.01, ^***^p < 0.001

Other significant factors were female, age, the number of chronic diseases, and college and above education for the outpatient visits. While they showed greater positive impacts at the higher quantiles, college and above education showed negative ones. As for inpatient visits and hospitalization days, age, being married, poor self-rated health, number of chronic diseases, and the CCI showed greater positive impacts at the higher quantiles [see Additional file 1: Appendix 5].

## Discussion

This study empirically examines the effect of PHI enrollment on the distribution of medical expenditure and health service utilization. Propensity score matching was used to moderate endogeneity. To account for skewed distribution with a large number of zero values, quantile regression was used to analyze medical expenditure, and quantile count regression was used to analyze health service utilization.

PHI enrollment had a significant positive effect on the lower quantiles (10th and 25th ) of outpatient expenditure distribution, typically for minor illnesses or preventive care. Previous studies [2, 10–12] showed that PHI increases outpatient expenditure. Our study provides further evidence that plausible moral hazard may exist along the lower quantiles of the outpatient expenditure distribution. PHI enrollment significantly increased outpatient service use in all quantiles except at the highest (90th ), peaking at the median (1.825, p < 0.001). This finding may explain the conflicting results in recent studies [10, 15, 16, 22, 24]. PHI effect was significant only within the group with fewer than 60 outpatient visits [16] and PHI significantly increased health service utilization among enrollees with mild diseases treatable in primary care, such as acute upper respiratory tract infections and upper gastrointestinal tract infections [31].

However, PHI enrollment showed no significant effect in any quantiles of inpatient expenditure. We found that while female enrollees spent more on inpatient expenditures than female non-enrollees, male enrollees spent less on them than male non-enrollees [see Additional file 1: Appendix 6]. Previous research has investigated gender differences in healthcare utilization [43]. Such research focused on women with a specific insurance, such as Medicaid, or with female-related health factors such as mammography [44], intrauterine devices [45], and endometriosis [46]. Disease severity among inpatients and physicians’ decision power over inpatients are possible explanatory factors; however, further study is warranted for the gender difference in inpatient costs among those enrolled in PHI.

Meanwhile, the PHI effect on the number of inpatient visits and hospitalization days increased along with higher quantiles. The insurance effect becomes more significant as individuals are hospitalized more frequently and for longer periods. Unlike fixed-benefit PHI, indemnity PHI may incentivize patients to stay longer at hospitals. When enrollees had both types of insurance, their hospital stay length increased by 65% compared to non-enrollees [47]. Further study is needed to examine the role played by the number or type of PHI.

It is possible for insurance companies not to select older individuals because they are more likely to suffer from illness, incurring higher costs. We conducted additional subgroup analysis excluding those aged 65 and over, and found similar results [see Additional file 1: Appendix 7].

South Korea has successfully implemented a national health insurance system, but the PHI enrollment rate has simultaneously increased over the last decade, with general household enrollment exceeding 90% as of 2019. On the one hand, PHI may supplement coverage gaps that national health insurance fails to fulfill, supporting medical accessibility. On the other hand, it may impose an additional burden on the financing of national health insurance due to unnecessarily increased health service utilization caused by moral hazard. Social inequality may have been exacerbated because of higher-income groups’ self-selection into PHI. It is essential to understand the distributional effect of PHI rather than the simple mean effect.

Our results indicate a need for an appropriate policy against moral hazard, focusing on the lower quantiles of outpatient expenditure and the upper quantiles of inpatient days. Recently, the Financial Services Commission—a Korean government organization with the statutory power to oversee financial policy—announced an amendment to the 4th generation indemnity insurance in July 2021. The amendment aimed to prevent excessive and unnecessary medical use by PHI enrollees, increase the rate of out-of-pocket expenses, and adjust health service utilization premiums. Specifically, while coverage for infertility and inherited cerebrovascular disease has been expanded, coverage for manual therapy and nutritional supplements has been restricted. We need an accustomed policy for outpatient services such as diagnosis, examination, and treatment of minor diseases to moderate the number of visits; and for inpatient services to moderate hospitalization days, for which PHI reimburses patients per day spent.

Furthermore, the productive effect of additional medical care uses, and expenditure driven by PHI, should be examined. In our study, PHI non-enrollees are more likely to be socially vulnerable and medically fragile with lower education, lower income, worse self-rated health, more diagnosed chronic diseases, and higher CCI scores. When it becomes cost-effective, national health insurance coverage can be expanded to enhance social equity, especially for the medically and socially vulnerable.

### Limitations

First, while propensity score matching was employed to moderate endogeneity in PHI enrollment, it was not possible to fully match unobserved characteristics due to data availability, such as individual risk behavior and health service preferences. Second, we chose to do a cross-sectional study using three years of panel data, driven by the need to utilize many variables with limited variation over time. Therefore, we could not utilize the timing variation between insurance enrollment and health service utilization. Third, we used the PHI enrollment status but not the PHI type. The KHPS offers information on PHI type, categorized as fixed benefits, indemnity, and mixed. Previous studies showed heterogeneous effects of PHI by type [7, 47–50]. We used PHI enrollment status as the primary variable of interest to ensure the moderate sample size and avoid recollection bias from the respondents in the data.

## Conclusion

We used the KHPS dataset to explore the effect of PHI enrollment on the distribution of medical expenditure and health service utilization in adults aged 19 and older in South Korea. Our study showed that PHI had a larger effect on the lower quantiles of outpatient costs, indicating that moral hazard was potentially present in costs that were likely to treat mild disease or medical check-ups. Furthermore, the PHI showed a greater effect on the higher quantiles of inpatient care utilization, measured by the number of visits and the days of hospitalization. Health service utilization may be concerned with the quantity of medical care and medical expenditure may have more to do with the quality of medical care. Government policies should take these heterogeneous distributional effects of private health insurance into account.

### Electronic supplementary material

Below is the link to the electronic supplementary material.

Supplementary Material 1

## Data Availability

The data that support the findings of this study are available from the Korea Health Panel Survey (https://www.khp.re.kr:444/eng/main.do) but restrictions apply to the availability of these data, which were used under license for the current study, and so are not publicly available. Data are however available from the authors (Wankyo Chung, wankyo@snu.ac.kr) upon reasonable request and with permission of the Korea Health Panel Survey.
