# Supplementary material for: Effects of private health insurance on medical expenditure and health service utilization in South Korea: a quantile regression analysis
Source: BMC Health Serv Res. 2023 Nov 7;23:1219. doi: 10.1186/s12913-023-10251-x (PMC10629166; doi:10.1186/s12913-023-10251-x)
Supplement: Supplementary file 1 — Supplementary Material 1 [file 12913_2023_10251_MOESM1_ESM.docx]

**Effects of private health insurance on medical expenditure and health service utilization in South Korea: a quantile regression analysis**

Kristine Namhee Kwon^1^, Wankyo Chung^2*^

^1^ Department of Health Policy and Management, Milken Institute School of Public Health, George Washington University, Washington DC, the United States of America.

^2^ Department of Public Health Sciences, Graduate School of Public Health, Seoul National University, Seoul, South Korea.

* Corresponding author

E-mail: [wankyo@snu.ac.kr](mailto:wankyo@snu.ac.kr)

Tel: 822-880-2285, Fax: 822-762-2888

Address: 1 Gwanak-ro, Gwanak-gu,

Graduate School of Public Health,

Seoul National University

**Supplementary information**

**Additional file 1. Appendices**

**Appendix 1.** Trends of NHI coverage rate and PHI enrollment rate 3

**Appendix 2.** Summary of previous literature controlling for endogeneity 4

**Appendix 3.** Description for variables used in the analysis 5

**Appendix 4.** Charlson’s comorbidity index with KHPS diagnostic codes 6

**Appendix 5.** Full regression tables

**Appendix 5a.** The effect of PHI on the outpatient costs 7

**Appendix 5b.** The effect of PHI on the inpatient costs 8

**Appendix 5c.** The effect of PHI on the total costs 9

**Appendix 5d.** The marginal effect of PHI on the outpatient visits 10

**Appendix 5e.** The marginal effect of PHI on the inpatient visits 11

**Appendix 5f.** The marginal effect of PHI on the inpatient days 12

**Appendix 6.** The effects of PHI on costs and services by gender 13

**Appendix 7.** The effects of PHI on costs and services for those under age 65 14

Appendix 1. Trends of NHI coverage rate and PHI enrollment rate

| **(%)** | **2012** | **2013** | **2014** | **2015** | **2016** | **2017** | **2018** | **2019** | **2020** |
| --- | --- | --- | --- | --- | --- | --- | --- | --- | --- |
| **NHI**^a^ **coverage** | 62.5 | 62.0 | 63.2 | 63.4 | 62.6 | 62.7 | 63.8 | 64.2 | 65.3 |
| **NHI**^a^ **enrollment rate** | 97.1 | 97.2 | 97.2 | 97.0 | 97.1 | 97.2 | 97.2 | 97.2 | 97.1 |
| **MA**^b^ **enrollment rate** | 2.9 | 2.8 | 2.8 | 3.0 | 2.9 | 2.8 | 2.8 | 2.8 | 2.9 |
| **PHI**^c^ **enrollment rate** | 69.8 | 70.1 | 72.2 | 72.0 | 74.0 | 75.4 | 78.2 | 78.0 | 78.6 |
| (General household) | 84.6 | 85.9 | 87.7 | 87.5 | 88.7 | 89.9 | 91.2 | 91.3 | 91.8 |
| (Low-income household) | 32.0 | 30.8 | 31.8 | 31.7 | 34.6 | 35.4 | 39.0 | 40.8 | 44.3 |
| **PHI**^c^ **enrollment count**^d^ | 3.0 | 2.9 | 3.2 | 3.2 | 3.3 | 3.4 | 3.5 | 4.9 | 4.9 |
| (General household) | 3.9 | 3.7 | 4.1 | 4.1 | 4.1 | 4.4 | 4.4 | 5.2 | 5.3 |
| (Low-income household) | 0.8 | 0.8 | 0.8 | 0.8 | 0.9 | 0.9 | 1.0 | 2.6 | 2.8 |

Note: ^a^NHI (National Health Insurance); ^b^MA (Medical Aid); ^c^PHI (Private Health Insurance); ^d^PHI enrollment count refers to the number of PHI enrollments; Data is from Health insurance Review & Assessment Service (National Health Insurance Statistical Yearbook), Ministry of Health and Welfare (National Health Insurance Service), and Korea Institute for Health and Social Affairs (Korea Welfare Panel Study).

Appendix 2. Summary of previous literature controlling for endogeneity

| **Methods for endogeneity** | **Studies** | **Main methods** | **Main results** | | | |
| --- | --- | --- | --- | --- | --- | --- |
|  |  |  | **Medical expenditure** | | **Health service utilization** | |
|  |  |  | **Outpatient** | **Inpatient** | **Outpatient** | **Inpatient** |
| Instrumental variable |  |  |  |  |  |  |
| Number of children, Private transfer | Kang et al. (2010) | Negative binomial | - | - | n/s | n/s |
| Number of insurance consultants in the residence, financial business occupation | You et al. (2014) | 2SLS | + | n/s | n/s | n/s |
| Personal insurance enrollment, number of cohabiting household members | You et al. (2018) | 2SLS | - | - | + | + |
| Household, marital status, education level | Choi (2016) | 2SLS | + | n/s | + | + |
| Panel method |  |  |  |  |  |  |
|  | You, Kwon (2020) | Panel logit | Female (n/s), Male (+) | Female (+), Male (n/s) | - | - |
|  | Yoon (2019) | CRE | - | - | + | n/s |
|  | Lee (2020) | Panel Poisson | - | - | + | + |
| Propensity score matching |  |  |  |  |  |  |
| Nearest-neighbor | Kim (2011) | ATT | - | - | + | + |
| Kernel | Kiil (2012) | ATT | - | - | n/s | n/s |
| Caliper | Lee, Nam (2013) | Panel regression | + | n/s | + | n/s |
| Nearest-neighbor | Jeon, Kwon (2013) | Two-part | + | n/s | n/s | n/s |
| Nearest-neighbor,  Kernel, Radius caliper | Thuong (2020) | ATT | - | - | + | + |

Note: (+) significantly increased, (-) significantly decreased, and (n/s) not significant; 2SLS (two-stage least squares); CRE (Correlated Random Effects); ATT (Average Treatment Effect on the Treated).

Appendix 3. Description of variables used in the analysis

| **Variables** | | | **Measures** |
| --- | --- | --- | --- |
| **Independent** | **Enrollment in private health insurance** | | Yes, No |
| **Dependent** | **Medical expenditure** | Outpatient medical expenditure (A) | |
|  |  | Inpatient medical expenditure (B) | |
|  |  | Total medical expenditure (A+B) | |
|  | **Healthcare utilization** | Number of outpatient visits | |
|  |  | Number of inpatient visits | |
|  |  | Days of hospitalization | |
| **Control** | **Predisposing factors** | Gender | Female, male |
|  |  | Age group | 20-29, 30-44, 45-59, 60-74, 75+ |
|  |  | Education | Primary, Middle, High, College and above |
|  |  | Marital status | Married, Single |
|  | **Enabling factors** | Residence | Capital area, other areas |
|  |  | Insurance | National Health Insurance, Medical Aid |
|  |  | Economically active | Yes, No |
|  |  | Yearly income level | 1^st^ quartile (lowest), 2^nd^ quartile, 3^rd^ quartile, 4^th^ quartile (highest) |
|  | **Illness**  **factors** | Self-rated health | Bad (Normal, Bad, Very Bad),  Good (Good, Very Good) |
|  |  | Number of chronic diseases | 0, 1, 2+ |
|  |  | Charlson’s comorbidity index | 0, 1, 2+ |

Note: Age, yearly income, number of chronic diseases, and Charlson’s comorbidity index were matched as categorical variables shown above and as continuous variables in the main analysis. Missing rates of “Self-rated health” in the original KHPS data were 4.46% (2016), 3.91% (2017), and 3.97% (2018). We excluded missing values from our analysis.

Appendix 4. Charlson’s comorbidity index with KHPS diagnostic codes

| **Diseases** | **KHPS KCD-6 Codes** | **CCI Scores** |
| --- | --- | --- |
| Myocardial infarction | I21, I22, I25 | 1 |
| Congestive heart failure | I50 | 1 |
| Peripheral vascular disease | I70-I79 | 1 |
| Cerebrovascular disease | I60-I69 | 1 |
| Dementia | F03, G30 | 1 |
| Chronic pulmonary disease | J41-J45, J47, J64 | 1 |
| Rheumatologic disease | M30-M36, M06 | 1 |
| Peptic ulcer disease | K25, K26 | 1 |
| Mild liver disease | B18, B19, K70-K77 | 1 |
| Diabetes without chronic complication | E10-E14 | 1 |
| Diabetes with chronic complication | None | 2 |
| Hemiplegia or paraplegia | G80, G81, G82 | 2 |
| Renal disease | N17-N19 | 2 |
| Any malignancy, including leukemia and lymphoma | C00-C41, C43, C45-C72, C74, C75, C81-C96 | 2 |
| Moderate or severe liver disease | None | 3 |
| Metastatic solid tumor | C76-C80 | 6 |
| HIV/AIDS | B20-B24 | 6 |

Note: CCI calculation is possible with the KHPS data since it utilizes the Korean Standard Classification of Diseases (KCD-6) based on the International Classification of Diseases (ICD-10). Diabetes was calculated in total and malignant neoplasm of the thyroid gland (C73) was excluded from the calculation because of excessive diagnostics issues in South Korea.

Appendix 5a. Full regression table of the effect of PHI on the outpatient cost

| **Outpatient cost** | **OLS** | **Quantile regression** | | | | | | |
| --- | --- | --- | --- | --- | --- | --- | --- | --- |
|  |  | **QR10** | **QR25** | **QR50** | **QR60** | **QR70** | **QR80** | **QR90** |
|  |  |  |  |  |  |  |  |  |
| PHI | 0.082^**^ | 0.149^**^ | 0.121^***^ | 0.061 | 0.043 | 0.022 | 0.028 | 0.038 |
|  | (0.026) | (0.047) | (0.036) | (0.031) | (0.030) | (0.032) | (0.029) | (0.037) |
|  |  |  |  |  |  |  |  |  |
| Year 2017 | 0.069^*^ | 0.096 | 0.015 | 0.058 | 0.086^*^ | 0.102^**^ | 0.048 | 0.070 |
|  | (0.032) | (0.056) | (0.044) | (0.037) | (0.037) | (0.036) | (0.034) | (0.043) |
|  |  |  |  |  |  |  |  |  |
| Year 2018 | 0.096^**^ | 0.123^*^ | 0.061 | 0.074 | 0.123^***^ | 0.109^**^ | 0.090^*^ | 0.164^***^ |
|  | (0.032) | (0.061) | (0.044) | (0.038) | (0.035) | (0.041) | (0.036) | (0.047) |
|  |  |  |  |  |  |  |  |  |
| Female | 0.190^***^ | 0.236^***^ | 0.245^***^ | 0.230^***^ | 0.184^***^ | 0.134^***^ | 0.111^**^ | 0.069 |
|  | (0.031) | (0.054) | (0.044) | (0.038) | (0.035) | (0.038) | (0.037) | (0.046) |
|  |  |  |  |  |  |  |  |  |
| Age | 0.011^***^ | 0.015^***^ | 0.015^***^ | 0.013^***^ | 0.011^***^ | 0.010^***^ | 0.008^***^ | 0.006^**^ |
|  | (0.001) | (0.002) | (0.002) | (0.002) | (0.002) | (0.002) | (0.002) | (0.002) |
|  |  |  |  |  |  |  |  |  |
| Junior | 0.100^*^ | 0.086 | 0.082 | 0.174^***^ | 0.123^***^ | 0.078 | 0.083 | 0.097 |
|  | (0.040) | (0.074) | (0.054) | (0.049) | (0.037) | (0.051) | (0.048) | (0.053) |
|  |  |  |  |  |  |  |  |  |
| Senior | -0.063 | -0.109 | -0.068 | -0.025 | -0.054 | -0.058 | -0.039 | -0.094 |
|  | (0.041) | (0.081) | (0.056) | (0.048) | (0.050) | (0.046) | (0.042) | (0.057) |
|  |  |  |  |  |  |  |  |  |
| College | -0.103 | -0.254^**^ | -0.161^*^ | -0.080 | -0.107 | -0.099 | -0.074 | -0.159^*^ |
|  | (0.054) | (0.087) | (0.080) | (0.064) | (0.064) | (0.066) | (0.055) | (0.076) |
|  |  |  |  |  |  |  |  |  |
| Married | 0.192^***^ | 0.140^**^ | 0.219^***^ | 0.253^***^ | 0.224^***^ | 0.233^***^ | 0.197^***^ | 0.147^**^ |
|  | (0.031) | (0.051) | (0.039) | (0.036) | (0.035) | (0.036) | (0.034) | (0.046) |
|  |  |  |  |  |  |  |  |  |
| Residence | -0.047 | -0.116^*^ | -0.073 | -0.044 | -0.015 | -0.004 | -0.000 | -0.060 |
|  | (0.030) | (0.056) | (0.042) | (0.038) | (0.034) | (0.037) | (0.033) | (0.044) |
|  |  |  |  |  |  |  |  |  |
| NHI | 0.272^**^ | 0.745^***^ | 0.526^***^ | 0.245 | 0.128 | -0.023 | -0.040 | -0.148 |
|  | (0.094) | (0.210) | (0.120) | (0.152) | (0.118) | (0.121) | (0.119) | (0.128) |
|  |  |  |  |  |  |  |  |  |
| Econ. Act. | 0.086^**^ | 0.158^**^ | 0.065 | 0.131^***^ | 0.062 | 0.027 | 0.021 | -0.016 |
|  | (0.029) | (0.051) | (0.040) | (0.034) | (0.033) | (0.035) | (0.032) | (0.041) |
|  |  |  |  |  |  |  |  |  |
| log (Inc.) | 0.104^***^ | 0.037 | 0.075^*^ | 0.131^***^ | 0.141^***^ | 0.160^***^ | 0.130^***^ | 0.148^***^ |
|  | (0.026) | (0.043) | (0.035) | (0.031) | (0.029) | (0.031) | (0.029) | (0.036) |
|  |  |  |  |  |  |  |  |  |
| Poor SRH | 0.270^***^ | 0.223^***^ | 0.238^***^ | 0.315^***^ | 0.302^***^ | 0.312^***^ | 0.291^***^ | 0.242^***^ |
|  | (0.033) | (0.064) | (0.045) | (0.037) | (0.037) | (0.036) | (0.035) | (0.049) |
|  |  |  |  |  |  |  |  |  |
| Diseasecount | 0.171^***^ | 0.237^***^ | 0.207^***^ | 0.170^***^ | 0.150^***^ | 0.134^***^ | 0.117^***^ | 0.079^***^ |
|  | (0.007) | (0.013) | (0.009) | (0.007) | (0.007) | (0.007) | (0.006) | (0.008) |
|  |  |  |  |  |  |  |  |  |
| CCI | 0.103^***^ | 0.014 | 0.069^**^ | 0.084^***^ | 0.076^***^ | 0.086^***^ | 0.082^***^ | 0.152^***^ |
|  | (0.018) | (0.032) | (0.024) | (0.016) | (0.019) | (0.016) | (0.016) | (0.028) |
|  |  |  |  |  |  |  |  |  |
| Constant | 3.213^***^ | 1.256^***^ | 2.051^***^ | 2.894^***^ | 3.492^***^ | 3.996^***^ | 4.775^***^ | 5.566^***^ |
|  | (0.210) | (0.371) | (0.285) | (0.277) | (0.250) | (0.261) | (0.246) | (0.299) |
| *N* | 8616 | 8616 | 8616 | 8616 | 8616 | 8616 | 8616 | 8616 |
| F | 194.133 |  |  |  |  |  |  |  |

Note: Standard errors in parentheses; ^*^ *p* < 0.05, ^**^ *p* < 0.01, ^***^ *p* < 0.001; PHI (Private health insurance); NHI (National health insurance enrollment); Econ. Act. (Economically active); Inc. (Yearly income); SRH (Self-rated health); Diseasecount (the number of chronic diseases diagnosed); CCI (Charlson’s comorbidity index).

Appendix 5b. Full regression table of the effect of PHI on the inpatient cost

| **Inpatient cost** | **OLS** | **Quantile regression** | | | | | | |
| --- | --- | --- | --- | --- | --- | --- | --- | --- |
|  |  | **QR10** | **QR25** | **QR50** | **QR60** | **QR70** | **QR80** | **QR90** |
|  |  |  |  |  |  |  |  |  |
| PHI | -0.044 | -0.018 | 0.021 | -0.017 | -0.016 | -0.056 | 0.016 | 0.040 |
|  | (0.056) | (0.051) | (0.072) | (0.065) | (0.079) | (0.074) | (0.058) | (0.084) |
|  |  |  |  |  |  |  |  |  |
| Year 2017 | -0.018 | -0.070 | 0.081 | 0.061 | 0.036 | 0.012 | -0.082 | -0.019 |
|  | (0.069) | (0.067) | (0.085) | (0.082) | (0.091) | (0.076) | (0.071) | (0.078) |
|  |  |  |  |  |  |  |  |  |
| Year 2018 | -0.008 | 0.088 | 0.105 | 0.009 | -0.044 | -0.034 | -0.179^**^ | -0.117 |
|  | (0.067) | (0.050) | (0.084) | (0.077) | (0.096) | (0.089) | (0.056) | (0.089) |
|  |  |  |  |  |  |  |  |  |
| Female | 0.001 | 0.158^*^ | -0.019 | -0.004 | -0.125 | -0.061 | -0.075 | -0.084 |
|  | (0.068) | (0.062) | (0.079) | (0.078) | (0.086) | (0.067) | (0.058) | (0.084) |
|  |  |  |  |  |  |  |  |  |
| Age | 0.005 | 0.002 | -0.001 | 0.002 | 0.001 | 0.004 | 0.008^**^ | 0.010^*^ |
|  | (0.003) | (0.004) | (0.003) | (0.003) | (0.003) | (0.003) | (0.002) | (0.004) |
|  |  |  |  |  |  |  |  |  |
| Junior | -0.023 | 0.103 | -0.058 | -0.019 | 0.020 | 0.020 | -0.049 | 0.096 |
|  | (0.084) | (0.082) | (0.100) | (0.123) | (0.136) | (0.138) | (0.090) | (0.083) |
|  |  |  |  |  |  |  |  |  |
| Senior | 0.018 | 0.059 | 0.000 | 0.020 | -0.089 | -0.088 | -0.102 | -0.012 |
|  | (0.090) | (0.061) | (0.120) | (0.096) | (0.123) | (0.123) | (0.099) | (0.140) |
|  |  |  |  |  |  |  |  |  |
| College | -0.029 | 0.141 | -0.030 | 0.004 | -0.051 | -0.164 | -0.215^*^ | -0.124 |
|  | (0.110) | (0.104) | (0.129) | (0.125) | (0.145) | (0.149) | (0.105) | (0.172) |
|  |  |  |  |  |  |  |  |  |
| Married | 0.106 | 0.192^*^ | 0.032 | 0.069 | 0.117 | 0.058 | 0.082 | 0.094 |
|  | (0.067) | (0.090) | (0.083) | (0.075) | (0.089) | (0.083) | (0.062) | (0.079) |
|  |  |  |  |  |  |  |  |  |
| Residence | 0.052 | 0.093 | 0.019 | 0.095 | 0.164^*^ | 0.003 | 0.048 | 0.017 |
|  | (0.067) | (0.052) | (0.090) | (0.087) | (0.081) | (0.071) | (0.069) | (0.075) |
|  |  |  |  |  |  |  |  |  |
| NHI | 0.089 | 0.085 | 0.057 | 0.012 | 0.017 | 0.017 | 0.144 | 0.079 |
|  | (0.143) | (0.213) | (0.160) | (0.208) | (0.192) | (0.143) | (0.151) | (0.103) |
|  |  |  |  |  |  |  |  |  |
| Econ. Act. | -0.107 | -0.168^**^ | -0.165^*^ | -0.125 | -0.114 | -0.063 | 0.008 | -0.035 |
|  | (0.064) | (0.056) | (0.078) | (0.071) | (0.085) | (0.078) | (0.058) | (0.078) |
|  |  |  |  |  |  |  |  |  |
| log (Inc.) | 0.031 | 0.017 | -0.012 | 0.023 | -0.014 | 0.056 | 0.045 | -0.034 |
|  | (0.056) | (0.051) | (0.072) | (0.067) | (0.077) | (0.070) | (0.040) | (0.077) |
|  |  |  |  |  |  |  |  |  |
| Poor SRH | 0.253^***^ | 0.132^**^ | 0.196^*^ | 0.319^***^ | 0.480^***^ | 0.444^***^ | 0.368^***^ | 0.337^***^ |
|  | (0.068) | (0.049) | (0.086) | (0.090) | (0.103) | (0.096) | (0.065) | (0.080) |
|  |  |  |  |  |  |  |  |  |
| Diseasecount | -0.008 | -0.011 | -0.000 | -0.030 | -0.018 | 0.005 | 0.004 | -0.012 |
|  | (0.014) | (0.014) | (0.018) | (0.016) | (0.022) | (0.023) | (0.014) | (0.012) |
|  |  |  |  |  |  |  |  |  |
| CCI | 0.122^***^ | 0.070^**^ | 0.073^*^ | 0.157^***^ | 0.105^*^ | 0.126^**^ | 0.153^***^ | 0.180^***^ |
|  | (0.031) | (0.024) | (0.033) | (0.042) | (0.043) | (0.046) | (0.036) | (0.042) |
|  |  |  |  |  |  |  |  |  |
| Constant | 6.921^***^ | 5.813^***^ | 6.968^***^ | 7.218^***^ | 7.808^***^ | 7.485^***^ | 7.528^***^ | 8.398^***^ |
|  | (0.453) | (0.557) | (0.530) | (0.519) | (0.545) | (0.444) | (0.302) | (0.605) |
| *N* | 1470 | 1470 | 1470 | 1470 | 1470 | 1470 | 1470 | 1470 |
| F | 5.005 |  |  |  |  |  |  |  |

Note: Standard errors in parentheses; ^*^ *p* < 0.05, ^**^ *p* < 0.01, ^***^ *p* < 0.001; PHI (Private health insurance); NHI (National health insurance enrollment); Econ. Act. (Economically active); Inc. (Yearly income); SRH (Self-rated health); Diseasecount (the number of chronic diseases diagnosed); CCI (Charlson’s comorbidity index).

Appendix 5c. Full regression table of the effect of PHI on the total cost

| **Total cost** | **OLS** | **Quantile regression** | | | | | | |
| --- | --- | --- | --- | --- | --- | --- | --- | --- |
|  |  | **QR10** | **QR25** | **QR50** | **QR60** | **QR70** | **QR80** | **QR90** |
|  |  |  |  |  |  |  |  |  |
| PHI | 0.111^***^ | 0.192^***^ | 0.159^***^ | 0.101^**^ | 0.085^*^ | 0.089^*^ | 0.103^*^ | 0.082 |
|  | (0.031) | (0.041) | (0.038) | (0.037) | (0.038) | (0.038) | (0.045) | (0.051) |
|  |  |  |  |  |  |  |  |  |
| Year 2017 | 0.053 | 0.119^*^ | 0.012 | 0.046 | 0.081 | 0.049 | 0.038 | 0.040 |
|  | (0.037) | (0.052) | (0.046) | (0.043) | (0.046) | (0.047) | (0.054) | (0.058) |
|  |  |  |  |  |  |  |  |  |
| Year 2018 | 0.077^*^ | 0.155^*^ | 0.070 | 0.082 | 0.094^*^ | 0.089 | 0.124^*^ | 0.021 |
|  | (0.038) | (0.061) | (0.048) | (0.047) | (0.047) | (0.049) | (0.052) | (0.064) |
|  |  |  |  |  |  |  |  |  |
| Female | 0.165^***^ | 0.214^***^ | 0.247^***^ | 0.185^***^ | 0.141^**^ | 0.102^*^ | 0.034 | 0.006 |
|  | (0.036) | (0.049) | (0.044) | (0.045) | (0.045) | (0.048) | (0.054) | (0.056) |
|  |  |  |  |  |  |  |  |  |
| Age | 0.013^***^ | 0.014^***^ | 0.015^***^ | 0.015^***^ | 0.014^***^ | 0.013^***^ | 0.009^***^ | 0.009^***^ |
|  | (0.002) | (0.002) | (0.002) | (0.002) | (0.002) | (0.002) | (0.002) | (0.002) |
|  |  |  |  |  |  |  |  |  |
| Junior | 0.102^*^ | 0.066 | 0.127^*^ | 0.174^***^ | 0.120^*^ | 0.146^*^ | 0.117^*^ | -0.003 |
|  | (0.048) | (0.057) | (0.065) | (0.053) | (0.060) | (0.063) | (0.057) | (0.067) |
|  |  |  |  |  |  |  |  |  |
| Senior | -0.140^**^ | -0.181^*^ | -0.110 | -0.064 | -0.121^*^ | -0.141^*^ | -0.256^***^ | -0.228^**^ |
|  | (0.048) | (0.073) | (0.057) | (0.058) | (0.060) | (0.056) | (0.071) | (0.085) |
|  |  |  |  |  |  |  |  |  |
| College | -0.118 | -0.318^***^ | -0.161^*^ | -0.097 | -0.083 | -0.067 | -0.198^*^ | -0.028 |
|  | (0.062) | (0.083) | (0.077) | (0.077) | (0.082) | (0.082) | (0.099) | (0.102) |
|  |  |  |  |  |  |  |  |  |
| Married | 0.227^***^ | 0.151^***^ | 0.236^***^ | 0.259^***^ | 0.266^***^ | 0.267^***^ | 0.224^***^ | 0.252^***^ |
|  | (0.036) | (0.045) | (0.043) | (0.043) | (0.044) | (0.045) | (0.050) | (0.059) |
|  |  |  |  |  |  |  |  |  |
| Residence | -0.107^**^ | -0.124^**^ | -0.122^**^ | -0.095^*^ | -0.065 | -0.106^*^ | -0.148^**^ | -0.097 |
|  | (0.035) | (0.047) | (0.042) | (0.042) | (0.043) | (0.042) | (0.047) | (0.060) |
|  |  |  |  |  |  |  |  |  |
| NHI | 0.107 | 0.448^***^ | 0.378^**^ | -0.065 | -0.201 | -0.204^*^ | -0.217 | -0.059 |
|  | (0.105) | (0.059) | (0.138) | (0.185) | (0.125) | (0.091) | (0.112) | (0.096) |
|  |  |  |  |  |  |  |  |  |
| Econ. Act. | 0.061 | 0.126^**^ | 0.073 | 0.072 | 0.010 | -0.006 | -0.042 | -0.036 |
|  | (0.033) | (0.046) | (0.042) | (0.041) | (0.042) | (0.043) | (0.051) | (0.055) |
|  |  |  |  |  |  |  |  |  |
| log (Inc.) | 0.127^***^ | 0.041 | 0.108^**^ | 0.158^***^ | 0.168^***^ | 0.169^***^ | 0.181^***^ | 0.175^***^ |
|  | (0.030) | (0.041) | (0.035) | (0.036) | (0.038) | (0.038) | (0.042) | (0.050) |
|  |  |  |  |  |  |  |  |  |
| Poor SRH | 0.422^***^ | 0.219^***^ | 0.335^***^ | 0.441^***^ | 0.469^***^ | 0.492^***^ | 0.474^***^ | 0.600^***^ |
|  | (0.040) | (0.049) | (0.051) | (0.045) | (0.049) | (0.046) | (0.058) | (0.073) |
|  |  |  |  |  |  |  |  |  |
| Diseasecount | 0.159^***^ | 0.241^***^ | 0.210^***^ | 0.162^***^ | 0.142^***^ | 0.118^***^ | 0.089^***^ | 0.063^***^ |
|  | (0.008) | (0.010) | (0.010) | (0.008) | (0.009) | (0.007) | (0.009) | (0.013) |
|  |  |  |  |  |  |  |  |  |
| CCI | 0.174^***^ | 0.063^*^ | 0.117^***^ | 0.153^***^ | 0.154^***^ | 0.169^***^ | 0.224^***^ | 0.270^***^ |
|  | (0.021) | (0.028) | (0.024) | (0.021) | (0.024) | (0.025) | (0.031) | (0.035) |
|  |  |  |  |  |  |  |  |  |
| Constant | 3.364^***^ | 1.603^***^ | 1.950^***^ | 3.069^***^ | 3.634^***^ | 4.203^***^ | 5.081^***^ | 5.669^***^ |
|  | (0.238) | (0.296) | (0.291) | (0.325) | (0.304) | (0.297) | (0.327) | (0.360) |
| *N* | 8634 | 8634 | 8634 | 8634 | 8634 | 8634 | 8634 | 8634 |
| F | 174.286 |  |  |  |  |  |  |  |

Note: Standard errors in parentheses; ^*^ *p* < 0.05, ^**^ *p* < 0.01, ^***^ *p* < 0.001; PHI (Private health insurance); NHI (National health insurance enrollment); Econ. Act. (Economically active); Inc. (Yearly income); SRH (Self-rated health); Diseasecount (the number of chronic diseases diagnosed); CCI (Charlson’s comorbidity index).

Appendix 5d. Full regression table of the marginal effect of PHI on the outpatient visit

| **Outpatient visit** | **NB/ZINB** | **Marginal effect of Quantile count regression** | | | | | | |
| --- | --- | --- | --- | --- | --- | --- | --- | --- |
|  |  | **QR10** | **QR25** | **QR50** | **QR60** | **QR70** | **QR80** | **QR90** |
|  |  |  |  |  |  |  |  |  |
| PHI | 2.283^***^ | 0.470^***^ | 0.873^***^ | 1.825^***^ | 1.648^***^ | 1.280^**^ | 1.478^*^ | 0.407 |
|  | (0.581) | (0.076) | (0.118) | (0.245) | (0.339) | (0.465) | (0.648) | (0.995) |
|  |  |  |  |  |  |  |  |  |
| Year 2017 | 0.771 | 0.021 | -0.182 | -0.031 | 0.221 | 0.476 | 0.209 | 0.037 |
|  | (0.697) | (0.089) | (0.148) | (0.317) | (0.371) | (0.538) | (0.864) | (1.119) |
|  |  |  |  |  |  |  |  |  |
| Year 2018 | 0.491 | -0.073 | -0.163 | -0.177 | 0.241 | 0.624 | 0.355 | -0.119 |
|  | (0.704) | (0.095) | (0.13) | (0.286) | (0.431) | (0.582) | (0.796) | (1.686) |
|  |  |  |  |  |  |  |  |  |
| Female | 6.341^***^ | 0.771^***^ | 1.696^***^ | 3.698^***^ | 3.867^***^ | 4.534^***^ | 5.486^***^ | 6.650^***^ |
|  | (0.674) | (0.095) | (0.158) | (0.286) | (0.404) | (0.510) | (0.671) | (1.304) |
|  |  |  |  |  |  |  |  |  |
| Age | 0.485^***^ | 0.055^***^ | 0.156^***^ | 0.325^***^ | 0.359^***^ | 0.371^***^ | 0.419^***^ | 0.503^***^ |
|  | (0.028) | (0.004) | (0.008) | (0.014) | (0.019) | (0.022) | (0.027) | (0.040) |
|  |  |  |  |  |  |  |  |  |
| Junior | 1.256 | 0.140 | 0.393 | 1.223^***^ | 0.884^*^ | 0.891 | 1.193 | 1.807 |
|  | (0.856) | (0.135) | (0.206) | (0.337) | (0.409) | (0.690) | (1.318) | (2.003) |
|  |  |  |  |  |  |  |  |  |
| Senior | -2.442^**^ | -0.454^**^ | -0.418^*^ | 0.098 | -0.826 | -1.630^**^ | -2.019^*^ | -4.142^*^ |
|  | (0.783) | (0.145) | (0.185) | (0.34) | (0.480) | (0.598) | (0.987) | (1.805) |
|  |  |  |  |  |  |  |  |  |
| College | -4.758^***^ | -0.795^***^ | -1.561^***^ | -2.541^***^ | -3.076^***^ | -3.844^***^ | -4.144^***^ | -6.197^**^ |
|  | (0.959) | (0.173) | (0.235) | (0.475) | (0.685) | (0.794) | (0.925) | (1.896) |
|  |  |  |  |  |  |  |  |  |
| Married | 7.406^***^ | 0.831^***^ | 2.193^***^ | 4.824^***^ | 5.231^***^ | 5.799^***^ | 7.442^***^ | 7.677^***^ |
|  | (0.692) | (0.078) | (0.147) | (0.300) | (0.369) | (0.523) | (0.680) | (1.165) |
|  |  |  |  |  |  |  |  |  |
| Residence | -2.118^**^ | -0.284^***^ | -0.637^***^ | -1.200^***^ | -1.098^**^ | -1.189^*^ | -1.423^*^ | -1.619 |
|  | (0.645) | (0.079) | (0.134) | (0.250) | (0.391) | (0.510) | (0.691) | (1.098) |
|  |  |  |  |  |  |  |  |  |
| NHI | -6.101^***^ | 0.393 | -0.533 | -2.932^*^ | -4.819 | -5.497^***^ | -5.872^*^ | -12.67^***^ |
|  | (1.735) | (0.29) | (0.683) | (1.167) | (3.404) | (1.176) | (2.340) | (2.979) |
|  |  |  |  |  |  |  |  |  |
| Econ. Act. | -0.035 | -0.085 | -0.451^***^ | 0.007 | -0.101 | 0.012 | -0.427 | 0.301 |
|  | (0.622) | (0.094) | (0.128) | (0.276) | (0.340) | (0.515) | (0.645) | (1.157) |
|  |  |  |  |  |  |  |  |  |
| log (Inc.) | 1.400^*^ | -0.072 | 0.147 | 0.578^*^ | 0.781^*^ | 1.058^*^ | 0.916 | 1.547 |
|  | (0.573) | (0.07) | (0.112) | (0.260) | (0.317) | (0.461) | (0.591) | (1.417) |
|  |  |  |  |  |  |  |  |  |
| Poor SRH | 6.165^***^ | 0.036 | 0.286 | 1.565^***^ | 2.709^***^ | 4.488^***^ | 7.149^***^ | 13.991^***^ |
|  | (0.733) | (0.097) | (0.147) | (0.358) | (0.529) | (0.832) | (1.436) | (1.886) |
|  |  |  |  |  |  |  |  |  |
| Diseasecount | 4.784^***^ | 0.398^***^ | 0.812^***^ | 1.725^***^ | 2.370^***^ | 3.185^***^ | 4.098^***^ | 5.908^***^ |
|  | (0.193) | (0.02) | (0.041) | (0.076) | (0.087) | (0.139) | (0.236) | (0.404) |
|  |  |  |  |  |  |  |  |  |
| CCI | -0.327 | 0.033 | -0.061 | -0.049 | -0.282 | -0.369 | -0.653 | -1.192 |
|  | (0.343) | (0.047) | (0.081) | (0.145) | (0.214) | (0.313) | (0.419) | (0.979) |
|  |  |  |  |  |  |  |  |  |
| *N* | 10654 | 10654 | 10654 | 10654 | 10654 | 10654 | 10654 | 10654 |
|  |  |  |  |  |  |  |  |  |

Note: Standard errors in parentheses; ^*^ *p* < 0.05, ^**^ *p* < 0.01, ^***^ *p* < 0.001; PHI (Private health insurance); NHI (National health insurance enrollment); Econ. Act. (Economically active); Inc. (Yearly income); SRH (Self-rated health); Diseasecount (the number of chronic diseases diagnosed); CCI (Charlson’s comorbidity index).

Appendix 5e. Full regression table of the marginal effect of PHI on the inpatient visit

| **Inpatient visit** | **NB/ZINB** | **Marginal effect of Quantile count regression** | | | | | | |
| --- | --- | --- | --- | --- | --- | --- | --- | --- |
|  |  | **QR10** | **QR25** | **QR50** | **QR60** | **QR70** | **QR80** | **QR90** |
|  |  |  |  |  |  |  |  |  |
| PHI | 0.109 | 0.004^*^ | 0.006^***^ | 0.013^***^ | 0.017^***^ | 0.020^***^ | 0.028^**^ | 0.076^**^ |
|  | (1.136) | (0.002) | (0.002) | (0.004) | (0.005) | (0.006) | (0.009) | (0.029) |
|  |  |  |  |  |  |  |  |  |
| Year 2017 | -0.097 | -0.001 | -0.002 | -0.003 | -0.004 | -0.005 | -0.008 | -0.035 |
|  | (1.011) | (0.002) | (0.002) | (0.004) | (0.006) | (0.007) | (0.011) | (0.032) |
|  |  |  |  |  |  |  |  |  |
| Year 2018 | -0.064 | -0.002 | -0.003 | -0.006 | -0.008 | -0.01 | -0.014 | -0.037 |
|  | (0.665) | (0.002) | (0.002) | (0.005) | (0.006) | (0.007) | (0.011) | (0.032) |
|  |  |  |  |  |  |  |  |  |
| Female | 0.082 | 0.002 | 0.003 | 0.005 | 0.007 | 0.01 | 0.016 | 0.055 |
|  | (0.857) | (0.002) | (0.002) | (0.004) | (0.005) | (0.007) | (0.011) | (0.033) |
|  |  |  |  |  |  |  |  |  |
| Age | 0.011 | 0.000^**^ | 0.000^***^ | 0.001^***^ | 0.001^***^ | 0.001^***^ | 0.002^***^ | 0.007^***^ |
|  | (0.110) | (0.000) | (0.000) | (0.000) | (0.000) | (0.000) | (0.000) | (0.002) |
|  |  |  |  |  |  |  |  |  |
| Junior | 0.183 | 0.001 | 0.003 | 0.007 | 0.008 | 0.006 | 0.000 | -0.031 |
|  | (0.094) | (0.003) | (0.003) | (0.006) | (0.008) | (0.010) | (0.015) | (0.044) |
|  |  |  |  |  |  |  |  |  |
| Senior | -0.121 | -0.007 | -0.010^**^ | -0.020^***^ | -0.025^***^ | -0.031^***^ | -0.049^***^ | -0.191^***^ |
|  | (0.065) | (0.008) | (0.004) | (0.005) | (0.007) | (0.008) | (0.012) | (0.036) |
|  |  |  |  |  |  |  |  |  |
| College | 0.012 | -0.003 | -0.003 | -0.006 | -0.007 | -0.007 | -0.011 | -0.105^*^ |
|  | (0.089) | (0.010) | (0.004) | (0.007) | (0.009) | (0.012) | (0.018) | (0.050) |
|  |  |  |  |  |  |  |  |  |
| Married | 0.325 | 0.007^*^ | 0.012^***^ | 0.024^***^ | 0.030^***^ | 0.038^***^ | 0.058^***^ | 0.182^***^ |
|  | (3.383) | (0.003) | (0.002) | (0.004) | (0.005) | (0.007) | (0.010) | (0.032) |
|  |  |  |  |  |  |  |  |  |
| Residence | -0.190 | -0.005 | -0.008^***^ | -0.016^***^ | -0.021^***^ | -0.026^***^ | -0.042^***^ | -0.140^***^ |
|  | (1.984) | (0.003) | (0.002) | (0.004) | (0.005) | (0.006) | (0.009) | (0.028) |
|  |  |  |  |  |  |  |  |  |
| NHI | -0.246 | -0.003 | -0.005 | -0.015 | -0.023 | -0.035 | -0.063 | -0.115 |
|  | (2.565) | (0.037) | (0.01) | (0.014) | (0.019) | (0.027) | (0.051) | (0.181) |
|  |  |  |  |  |  |  |  |  |
| Econ. Act. | -0.041 | 0.000 | 0.000 | 0.000 | 0.000 | -0.001 | -0.007 | -0.063 |
|  | (0.428) | (0.002) | (0.002) | (0.004) | (0.005) | (0.007) | (0.01) | (0.034) |
|  |  |  |  |  |  |  |  |  |
| log (Inc.) | 0.022 | 0.002 | 0.004^*^ | 0.008^*^ | 0.011^*^ | 0.013^*^ | 0.017^*^ | 0.078^**^ |
|  | (0.234) | (0.001) | (0.002) | (0.004) | (0.004) | (0.006) | (0.009) | (0.028) |
|  |  |  |  |  |  |  |  |  |
| Poor SRH | 0.626 | 0.009^**^ | 0.019^***^ | 0.042^***^ | 0.053^***^ | 0.075^***^ | 0.158^***^ | 0.338^***^ |
|  | (6.522) | (0.003) | (0.003) | (0.007) | (0.008) | (0.012) | (0.028) | (0.080) |
|  |  |  |  |  |  |  |  |  |
| Diseasecount | 0.047 | 0.001^*^ | 0.002^***^ | 0.004^***^ | 0.005^***^ | 0.007^***^ | 0.009^***^ | 0.020^**^ |
|  | (0.491) | (0.000) | (0.001) | (0.001) | (0.001) | (0.002) | (0.003) | (0.007) |
|  |  |  |  |  |  |  |  |  |
| CCI | 0.342 | 0.004^**^ | 0.006^***^ | 0.015^***^ | 0.021^***^ | 0.031^***^ | 0.055^***^ | 0.131^***^ |
|  | (3.562) | (0.001) | (0.001) | (0.003) | (0.003) | (0.005) | (0.007) | (0.021) |
|  |  |  |  |  |  |  |  |  |
| *N* | 10654 | 10654 | 10654 | 10654 | 10654 | 10654 | 10654 | 10654 |
|  |  |  |  |  |  |  |  |  |

Note: Standard errors in parentheses; ^*^ *p* < 0.05, ^**^ *p* < 0.01, ^***^ *p* < 0.001; PHI (Private health insurance); NHI (National health insurance enrollment); Econ. Act. (Economically active); Inc. (Yearly income); SRH (Self-rated health); Diseasecount (the number of chronic diseases diagnosed); CCI (Charlson’s comorbidity index).

Appendix 5f. Full regression table of the marginal effect of PHI on the inpatient days

| **Inpatient days** | **NB/ZINB** | **Marginal effect of Quantile count regression** | | | | | | |
| --- | --- | --- | --- | --- | --- | --- | --- | --- |
|  |  | **QR10** | **QR25** | **QR50** | **QR60** | **QR70** | **QR80** | **QR90** |
|  |  |  |  |  |  |  |  |  |
| PHI | 0.045 | 0.004^*^ | 0.006^***^ | 0.013^***^ | 0.017^***^ | 0.024^**^ | 0.050^*^ | 0.321^**^ |
|  | (0.140) | (0.002) | (0.002) | (0.004) | (0.005) | (0.008) | (0.019) | (0.119) |
|  |  |  |  |  |  |  |  |  |
| Year 2017 | -0.166 | -0.001 | -0.002 | -0.003 | -0.004 | -0.007 | -0.008 | -0.064 |
|  | (0.168) | (0.002) | (0.002) | (0.005) | (0.006) | (0.009) | (0.021) | (0.135) |
|  |  |  |  |  |  |  |  |  |
| Year 2018 | -0.514^**^ | -0.002 | -0.003 | -0.006 | -0.008 | -0.011 | -0.01 | -0.125 |
|  | (0.175) | (0.002) | (0.002) | (0.005) | (0.006) | (0.01) | (0.023) | (0.119) |
|  |  |  |  |  |  |  |  |  |
| Female | -0.026 | 0.002 | 0.003 | 0.005 | 0.007 | 0.009 | 0.014 | 0.259 |
|  | (0.155) | (0.002) | (0.002) | (0.004) | (0.006) | (0.009) | (0.021) | (0.147) |
|  |  |  |  |  |  |  |  |  |
| Age | 0.026^***^ | 0.000^**^ | 0.000^***^ | 0.001^***^ | 0.001^***^ | 0.001^*^ | 0.002^*^ | 0.038^***^ |
|  | (0.007) | (0.000) | (0.000) | (0.000) | (0.000) | (0.000) | (0.001) | (0.007) |
|  |  |  |  |  |  |  |  |  |
| Junior | -0.101 | 0.001 | 0.003 | 0.007 | 0.009 | 0.011 | 0.011 | 0.153 |
|  | (0.235) | (0.003) | (0.003) | (0.007) | (0.009) | (0.015) | (0.036) | (0.203) |
|  |  |  |  |  |  |  |  |  |
| Senior | -0.642^**^ | -0.007 | -0.010^**^ | -0.021^***^ | -0.026^***^ | -0.038^***^ | -0.088^***^ | -0.660^***^ |
|  | (0.204) | (0.008) | (0.004) | (0.005) | (0.007) | (0.011) | (0.025) | (0.158) |
|  |  |  |  |  |  |  |  |  |
| College | -0.636^**^ | -0.003 | -0.003 | -0.006 | -0.006 | -0.007 | -0.020 | -0.433^*^ |
|  | (0.234) | (0.01) | (0.004) | (0.008) | (0.01) | (0.015) | (0.036) | (0.217) |
|  |  |  |  |  |  |  |  |  |
| Married | 0.121 | 0.007^*^ | 0.012^***^ | 0.024^***^ | 0.031^***^ | 0.044^***^ | 0.079^***^ | 0.584^***^ |
|  | (0.161) | (0.003) | (0.002) | (0.004) | (0.006) | (0.009) | (0.022) | (0.139) |
|  |  |  |  |  |  |  |  |  |
| Residence | -0.814^***^ | -0.005 | -0.008^***^ | -0.016^***^ | -0.022^***^ | -0.033^***^ | -0.074^***^ | -0.568^***^ |
|  | (0.170) | (0.003) | (0.002) | (0.004) | (0.005) | (0.008) | (0.018) | (0.128) |
|  |  |  |  |  |  |  |  |  |
| NHI | -0.842^*^ | -0.003 | -0.005 | -0.017 | -0.033 | -0.08 | -0.126 | -1.04 |
|  | (0.407) | (0.037) | (0.01) | (0.016) | (0.027) | (0.055) | (0.186) | (1.387) |
|  |  |  |  |  |  |  |  |  |
| Econ. Act. | -0.233 | 0.000 | 0.000 | 0.001 | 0.000 | -0.002 | -0.011 | -0.264^*^ |
|  | (0.158) | (0.002) | (0.002) | (0.004) | (0.006) | (0.009) | (0.022) | (0.128) |
|  |  |  |  |  |  |  |  |  |
| log (Inc.) | -0.006 | 0.002 | 0.004^**^ | 0.008^*^ | 0.011^*^ | 0.016^*^ | 0.023 | 0.263^*^ |
|  | (0.144) | (0.001) | (0.002) | (0.004) | (0.005) | (0.007) | (0.017) | (0.128) |
|  |  |  |  |  |  |  |  |  |
| Poor SRH | 1.202^***^ | 0.009^**^ | 0.019^***^ | 0.042^***^ | 0.056^***^ | 0.119^***^ | 0.677^**^ | 1.520^***^ |
|  | (0.201) | (0.003) | (0.003) | (0.007) | (0.009) | (0.027) | (0.222) | (0.353) |
|  |  |  |  |  |  |  |  |  |
| Diseasecount | 0.061 | 0.001^*^ | 0.002^***^ | 0.004^***^ | 0.006^***^ | 0.010^***^ | 0.026^***^ | 0.097^**^ |
|  | (0.037) | (0.000) | (0.001) | (0.001) | (0.002) | (0.003) | (0.007) | (0.031) |
|  |  |  |  |  |  |  |  |  |
| CCI | 0.306^***^ | 0.004^**^ | 0.006^***^ | 0.016^***^ | 0.024^***^ | 0.050^***^ | 0.101^***^ | 0.388^***^ |
|  | (0.085) | (0.001) | (0.001) | (0.003) | (0.005) | (0.008) | (0.020) | (0.082) |
|  |  |  |  |  |  |  |  |  |
| *N* | 10654 | 10654 | 10654 | 10654 | 10654 | 10654 | 10654 | 10654 |
|  |  |  |  |  |  |  |  |  |

Note: Standard errors in parentheses; ^*^ *p* < 0.05, ^**^ *p* < 0.01, ^***^ *p* < 0.001; PHI (Private health insurance); NHI (National health insurance enrollment); Econ. Act. (Economically active); Inc. (Yearly income); SRH (Self-rated health); Diseasecount (the number of chronic diseases diagnosed); CCI (Charlson’s comorbidity index).

Appendix 6. The effects of PHI on costs and services by gender

| **Cost** | **OLS regression** | **Quantile regression** | | | | | | |
| --- | --- | --- | --- | --- | --- | --- | --- | --- |
|  |  | **0.10** | **0.25** | **0.50** | **0.60** | **0.70** | **0.80** | **0.90** |
| **Female** |  |  |  |  |  |  |  |  |
| **Out.** | -0.055 | -0.096 | -0.045 | 0.003 | 0.012 | 0.032 | -0.048 | -0.178^*^ |
| (N=8616) | (0.054) | (0.091) | (0.076) | (0.067) | (0.062) | (0.064) | (0.059) | (0.077) |
| **In.** | 0.349^**^ | 0.268^*^ | 0.302^*^ | 0.350^*^ | 0.369^*^ | 0.309^*^ | 0.227^*^ | 0.465^**^ |
| (N=1470) | (0.115) | (0.126) | (0.151) | (0.150) | (0.152) | (0.140) | (0.102) | (0.158) |
| **Total** | -0.076 | -0.109 | -0.129 | -0.092 | -0.023 | -0.059 | -0.104 | -0.064 |
| (N=8634) | (0.063) | (0.086) | (0.077) | (0.076) | (0.077) | (0.084) | (0.093) | (0.101) |
| **Male** |  |  |  |  |  |  |  |  |
| **Out.** | 0.055 | 0.096 | 0.045 | -0.003 | -0.012 | -0.032 | 0.048 | 0.178^*^ |
| (N=8616) | (0.054) | (0.091) | (0.076) | (0.067) | (0.062) | (0.064) | (0.059) | (0.077) |
| **In.** | -0.349^**^ | -0.268^*^ | -0.302^*^ | -0.350^*^ | -0.369^*^ | -0.309^*^ | -0.227^*^ | -0.465^**^ |
| (N=1470) | (0.115) | (0.126) | (0.151) | (0.150) | (0.152) | (0.140) | (0.102) | (0.158) |
| **Total** | 0.076 | 0.109 | 0.129 | 0.092 | 0.023 | 0.059 | 0.104 | 0.064 |
| (N=8634) | (0.063) | (0.086) | (0.077) | (0.076) | (0.077) | (0.084) | (0.093) | (0.101) |
|  |  |  |  |  |  |  |  |  |
| **Service** | **NB/ZINB** | **Quantile count regression** | | | | | | |
|  |  | **0.10** | **0.25** | **0.50** | **0.60** | **0.70** | **0.80** | **0.90** |
| **Female** |  |  |  |  |  |  |  |  |
| **Out. visits** | -2.000 | 0.261 | 0.156 | 0.090 | 0.046 | -0.882 | -1.745 | -4.131 |
| **(**N=10654) | (1.176) | (0.170) | (0.263) | (0.523) | (0.660) | (0.873) | (1.207) | (2.341) |
| **In. visits** | 0.072 | -0.004 | -0.006 | -0.013 | -0.016 | -0.018 | -0.028 | -0.429 |
| **(**N=10654) | (0.299) | (0.004) | (0.004) | (0.007) | (0.008) | (0.011) | (0.017) | (0.236) |
| **In. days** | -0.311 | -0.004 | -0.007 | -0.014 | -0.018^*^ | -0.023 | -0.044 | -0.168 |
| (N=10654) | (0.289) | (0.004) | (0.004) | (0.007) | (0.009) | (0.015) | (0.033) | (0.221) |
| **Male** |  |  |  |  |  |  |  |  |
| **Out. visits** | 2.000 | -0.238 | -0.153 | -0.090 | -0.045 | 0.904 | 1.815 | 4.384 |
| **(**N=10654) | (1.176) | (0.141) | (0.251) | (0.518) | (0.659) | (0.918) | (1.299) | (2.367) |
| **In. visits** | -0.072 | 0.204 | 0.241 | 0.228 | 0.233 | 0.224 | 0.262 | 0.429 |
| **(**N=10654) | (0.098) | (0.199) | (0.141) | (0.127) | (0.129) | (0.139) | (0.164) | (0.236) |
| **In. days** | 0.311 | 0.204 | 0.242 | 0.237 | 0.261 | 0.265 | 0.302 | 0.254 |
| (N=10654) | (0.289) | (0.200) | (0.141) | (0.129) | (0.138) | (0.178) | (0.244) | (0.354) |

Note: Numbers in parentheses represent the robust standard errors; Other covariates were not displayed on the table; ^*^ *p* < 0.05, ^**^ *p* < 0.01, ^***^ *p* < 0.001; Negative binomial regression (NB) is used for outpatient visits, and Zero-inflated negative binomial regression (ZINB) is used for inpatient visits and inpatient days; Marginal effects are presented for the health service utilization variables; Out. (Outpatient); In. (Inpatient).

Appendix 7. The effects of PHI on costs and services for those under age 65

| **Cost** | **OLS regression** | **Quantile regression** | | | | | | |
| --- | --- | --- | --- | --- | --- | --- | --- | --- |
|  |  | **0.10** | **0.25** | **0.50** | **0.60** | **0.70** | **0.80** | **0.90** |
| **Out.** | 0.135^**^ | 0.225^**^ | 0.215^***^ | 0.096 | 0.048 | 0.095 | 0.070 | 0.093 |
| (N=3763) | (0.044) | (0.070) | (0.061) | (0.054) | (0.053) | (0.056) | (0.053) | (0.067) |
| **In.** | -0.051 | 0.096 | -0.003 | -0.016 | 0.012 | -0.007 | -0.044 | 0.070 |
| (N=479) | (0.096) | (0.138) | (0.103) | (0.105) | (0.094) | (0.101) | (0.085) | (0.107) |
| **Total** | 0.140^**^ | 0.229^**^ | 0.241^***^ | 0.121^*^ | 0.119 | 0.143^*^ | 0.102 | 0.081 |
| (N=3777) | (0.050) | (0.074) | (0.060) | (0.061) | (0.065) | (0.065) | (0.083) | (0.074) |
|  |  |  |  |  |  |  |  |  |
| **Service** | **NB/ZINB** | **Quantile count regression** | | | | | | |
|  |  | **0.10** | **0.25** | **0.50** | **0.60** | **0.70** | **0.80** | **0.90** |
| **Out. visits** | 2.613^***^ | 0.148^***^ | 0.354^***^ | 0.901^***^ | 1.290^***^ | 0.913^*^ | 1.023 | 1.473^*^ |
| (N=5625) | (0.558) | (0.028) | (0.068) | (0.188) | (0.304) | (0.428) | (0.560) | (0.724) |
| **In. visits** | -0.000 | 0.076 | 0.185 | 0.234^*^ | 0.231^*^ | 0.230^*^ | 0.237 | 0.305 |
| (N=5625) | (0.072) | (0.221) | (0.124) | (0.118) | (0.114) | (0.115) | (0.126) | (0.178) |
| **In. days** | -0.053 | 0.085 | 0.186 | 0.243^*^ | 0.237 | 0.225 | 0.248 | 0.241 |
| (N=5625) | (0.104) | (0.243) | (0.125) | (0.123) | (0.121) | (0.130) | (0.159) | (0.264) |

Note: Numbers in parentheses represent the robust standard errors; Other covariates were not displayed on the table; ^*^ *p* < 0.05, ^**^ *p* < 0.01, ^***^ *p* < 0.001; Negative binomial regression (NB) is used for outpatient visits, and Zero-inflated negative binomial regression (ZINB) is used for inpatient visits and inpatient days; Marginal effects are presented for the health service utilization variables; Out. (Outpatient); In. (Inpatient).
